# The Relationship Between Fetal Health Anxiety, Spiritual Well-Being, and Perceived Social Support in High-Risk Pregnant Women in Türkiye

**DOI:** 10.1007/s10943-025-02288-5

**Published:** 2025-03-07

**Authors:** Sibel Kiyak

**Affiliations:** Department of Obstetrics and Gynecology Nursing, Seydişehir Kamil Akkanat Faculty of Health Sciences, Necmettin Erbakan University, Anabağlar District, Prof. Dr. Necmettin Erbakan Street No:19/3, 42370 Seydişehir, Konya Türkiye

**Keywords:** Anxiety, High-risk pregnancy, Social support, Spiritual well-being

## Abstract

Fetal health anxiety during pregnancy is considered one of the most common and intense types of anxiety experienced. This study was conducted to determine the relationship between fetal health anxiety, spiritual well-being, and perceived social support levels in hospitalized pregnant women with high-risk pregnancies. This descriptive study was carried out between March and August 2024 with 175 pregnant women diagnosed with high-risk pregnancies in the obstetrics department of a medical faculty hospital in Türkiye. Data were collected using a descriptive characteristics form, the fetal health anxiety inventory (FHAI), the three-factor spiritual well-being scale (SWBS), and the multidimensional perceived social support scale (MSPSS). Hospitalized women with high-risk pregnancies reported low levels of fetal health anxiety and high levels of perceived social support. It was found that pregnant women with higher educational levels and those using medication regularly had higher perceived social support levels. It was determined that the anomie subdimension of the SWBS significantly differed based on pregnancy status, while the transcendence and harmony with nature subdimensions showed significant differences based on income level. An increase in the harmony with nature score resulted in a 0.282-unit increase in the perceived social support score (*β* = 0.282; *p* = 0.005). Similarly, an increase in the anomie subdimension score led to a 0.211-unit increase in the fetal health anxiety score (*β* = 0.211; *p* = 0.005). Health professionals, in addition to providing medical interventions in high-risk pregnancies, can offer holistic care by strengthening spiritual care and social support networks.

## Introduction

Pregnancy incorporates a wide range of emotions and experiences. Concerns regarding the health of the fetus are a significant dimension of this period. In particular, these concerns become more pronounced in cases of high-risk pregnancies, potentially exacerbating the stress experienced by pregnant women (Baltacı & Vatansever, 2024; Isaacs & Andipatin, 2020). In such pregnancies, maintaining a healthy course may require lifestyle adjustments, medical support, and even hospitalization. However, these interventions can lead to substantial lifestyle changes, including distancing the pregnant woman from her family and limiting her prior daily activities (Palma et al., 2021). This process often intensifies the focus on the fetus’s health, thereby heightening fetal health anxiety. Fetal health anxiety refers to the expectant mother’s worries concerning the health of her fetus (Reiser, 2019). In the literature, fetal health anxiety is considered one of the most prevalent and intense concerns during pregnancy (Reiser & Wright, 2019). Particularly in high-risk pregnancies, this anxiety can subject pregnant women to both physically and emotionally challenging experiences. At this point, spiritual well-being and perceived social support may play a significant role in managing the anxiety experienced by pregnant women (Hosaini et al., 2023).

Spiritual well-being refers to an individual’s ability to focus on their lives in line with adding value, meaning, and purpose. This attribute not only improves psychological functioning but also promotes stability, tranquility, and coordination in life. Additionally, it fosters closer relationships with oneself, a higher power, society, and the environment (Tirgari et al., 2022). Previous studies indicate that spirituality is effective in improving tolerance toward life challenges, enhancing life satisfaction, and coping with adversities (Akbarzadeh & Ahmadinezhad, 2019; Nourimand et al., 2020). Particularly in high-risk pregnancies, spiritual well-being was reported to positively influence stress management. For instance, a study carried out on pregnant women diagnosed with preeclampsia revealed that a higher level of spiritual well-being is related to reduce perceived stress (Akbarzadeh & Ahmadinezhad, 2019). Similarly, spiritual well-being is reported to improve coping skills and reduce fetal health anxiety in high-risk pregnancies (Chehrazi et al., 2021).

Social support refers to the emotional, material, or knowledge support that individuals receive through their connections with family members, friends, groups, and communities (Ozbay et al., 2007). Social support is a significant factor that positively affects women’s health, particularly during pregnancy, childbirth, and the postpartum period (Akbari et al., 2020). Strong social support networks reduce psychological distress during pregnancy and improve coping skills for managing stress (Bedaso et al., 2021; Karakoç et al., 2023). Moreover, it was emphasized that pregnant women with robust social support networks report higher levels of spiritual well-being (Akbarzadeh & Ahmadinezhad, 2019), which, in turn, may contribute to alleviating fetal health anxiety.

Interventions aiming to reduce fetal health anxiety in high-risk pregnancies play a crucial role in managing this process effectively. Despite existing studies on pregnancy-related anxiety, it is evident that the relationship between spiritual well-being, social support, and fetal health anxiety in high-risk pregnancies requires further detailed study. In this context, this study aims to analyze the relationship between fetal health anxiety, spiritual well-being, and perceived social support in high-risk pregnancies. This study provides a valuable framework for developing holistic and effective approaches to managing anxiety encountered in high-risk pregnancies by addressing these factors collectively.

## Research Questions

- What are the levels of fetal health anxiety, spiritual well-being, and perceived social support among hospitalized pregnant women diagnosed with high-risk pregnancies?
- Is there a relationship between fetal health anxiety, spiritual well-being, and perceived social support levels in hospitalized pregnant women diagnosed with high-risk pregnancies?

## Materials and Methods

### Study Design

The present study has a descriptive and correlational design.

### Sample and Participants

The study population consisted of pregnant women diagnosed with high-risk pregnancies and receiving treatment in the obstetrics ward of a university hospital between March and August 2024. The sample size was determined using the G*Power 3.1.9.4 program. The effect sizes used in the calculation were based on prior studies: *d* = 0.34 from Gökbulut et al. (2024) using the fetal health anxiety inventory (FHAI), *d* = 0.23 from Bilgiç and Çıtak Bilgin (2021) utilizing the spiritual well-being scale (SWBS), and *d* = 0.22 from Sarmasti et al. (2019) employing the multidimensional perceived social support scale (MSPSS). Using the smallest effect size (*d* = 0.22) with a 95% confidence level, 80% power, the required sample size was calculated as 169 participants. This study was completed with 175 participants. Inclusion criteria included pregnant women who are 18 years or older, able to read and write in Turkish, in any trimester, and diagnosed with a high-risk pregnancy (e.g., preeclampsia, placenta previa, preterm labor, gestational diabetes mellitus, abortion, etc.) by a physician. Pregnant women with a psychiatric diagnosis (self-reported) were excluded from this study.

### Measures

Data collection was performed using the following instruments.

#### Descriptive Characteristics Form

This form consisted of items designed to collect sociodemographic and obstetric characteristics.

#### Fetal Health Anxiety Inventory (FHAI)

Introduced by Reiser and Wright (2019) and adapted into Turkish by Gökbulut et al. (2024), this scale measures anxiety related to fetal health in pregnant women. The scale does not have subscales and consists of 14 items. Every item is scored on a 4-point Likert scale, ranging between 0 (no symptoms) and 3 (severe symptoms). Considering the total scores ranging between 0 and 42, a higher score indicates a higher level of fetal health anxiety. The Cronbach’s α coefficient for this scale was reported to be 0.85 (Gökbulut et al., 2024), while it was calculated as 0.846 in the present study.

#### Three-Factor Spiritual Well-Being Scale (SWBS)

Introduced by Ekşi and Kardaş (2017), this scale evaluates an individual’s ability to attribute meaning to life in individual, spiritual, and social dimensions and to determine the quality of this process. Due to the presence of other scales with similar names in the literature, the scale was renamed as the “Three-Factor Spiritual Well-Being Scale” (Kardaş, 2019). This scale is a 5-point Likert-type instrument consisting of three subscales: transcendence, harmony with nature, and anomie. Items in the anomie subscale are reverse-scored for the total score calculation. Considering the total scores ranging between 29 and 145, a higher score indicates a better spiritual well-being. The Cronbach’s alpha coefficient for the total scale score was calculated as 0.886, with values of 0.953 for transcendence, 0.864 for harmony with nature, and 0.853 for anomie (Ekşi & Kardaş, 2017). In the present study, however, Cronbach’s alpha value was found to be 0.794 for the total score, with subdimension values of 0.855 for transcendence, 0.685 for harmony with nature, and 0.773 for anomie. Given the risk of contamination of spiritual assessment tools with indicators of mental and social health (Koenig & Carey, 2024), analyses were conducted at the subdimension level of the scale.

#### Multidimensional Social Support Scale (MSPSS)

Introduced by Zimet et al. (1988) and adapted into Turkish by Eker and Arkar (1995), this 12-item Likert-type scale measures perceived social support and is scored between 1 (strongly disagree) and 7 (strongly agree). This scale includes three subscales: family support, friend support, and support from a significant other. Higher total scores indicate higher levels of perceived social support. The Cronbach’s alpha coefficient for the MSPSS was reported to be 0.78–0.92 (Eker & Arkar, 1995), and it was calculated to be 0.904 in this study.

### Data Collection

The data were collected by the researcher through face-to-face interviews. Each interview lasted approximately 20 min.

### Ethical Considerations

Before the study, ethical approval (date: 2024; no: 693) and institutional permission were obtained. Necessary permissions have been obtained from the authors who developed and/or adapted the scales in to Turkish. Before data collection, the purpose of this study was explained to the pregnant participants, and informed consent was obtained.

### Data Analysis

The data analysis was performed using IBM SPSS version 29 and IBM AMOS V24 program. The normality of the distribution was examined with the Shapiro–Wilk and the Kolmogorov–Smirnov tests. Comparisons of the data were performed using the Mann–Whitney *U* and the Kruskal–Wallis *H* tests. The relationships between continuous variables were analyzed using Spearman’s correlation test. In the study, structural equation modeling (SEM) analysis was used to examine the relationships between variables. In the study, fit indices were evaluated based on the criteria that the CMIN/DF value should be between 3 and 5, goodness of fit index (GFI), comparative fit index (CFI), and adjusted goodness of fit index (AGFI) should be greater than 0.90, and root mean squared error of approximation (RMSEA) should be less than 0.08 (Gürbüz, 2019). Quantitative data were presented as mean ± standard deviation and median (minimum–maximum), whereas categorical data were reported as frequencies and percentages. Statistical significance was set at *p* < 0.05.

## Results

The mean age of the 175 pregnant participants was 27.98 ± 6.01 years. Among the participants, 35.4% had completed primary education, 15.4% were employed, and 62.3% reported an income equal to their expenses. About 33.1% of the participants had chronic illness, 74% were taking medication continuously during pregnancy. It was determined that 69.7% of pregnancies were planned, and 41.7% of the participants were primiparous (Table 1).

**Table 1:** Comparison of fetal health anxiety inventory, subdimensions of the spiritual well-being scale, and multidimensional scale of perceived social support scores according to variables

Variables	*n* (%)	FHAI	MSPSS	Anomie	Transcendence	Harmony with nature
		Median (Min–Max)	Median (Min–Max)	Median (Min–Max)	Median (Min–Max)	Median (Min–Max)
*Education*						
Primary education	62 (35.4)	16 (5–41)	60 (33–84)^a^	17 (7–35)	71 (30–75)	33 (14–35)
High school	59 (33.7)	15 (4–35)	70 (12–84)^ab^	15 (7–35)	72 (52–75)	32 (18–35)
University or higher	54 (30.9)	16 (5–33)	71 (21–84)^b^	15 (7–26)	73 (47–75)	33 (26–35)
Test/*p* value		1.676/0.433^x^	6.720/**0.035**^**x**^	3.162/0.206^x^	0.881/0.644^x^	1.632/0.442^x^
*Employment status*						
No	148 (84.6)	16 (4–41)	68 (33–84)	15 (7–35)	72 (30–75)	33 (14–35)
Yes	27 (15.4)	14 (6–32)	66 (12–83)	15 (9–25)	72 (52–75)	33 (26–35)
Test/*p* value		1674/0.180^y^	1764/0.333^y^	1984.5/0.955^y^	1915.5/0.731^y^	1985/0.957^y^
*Income status*						
Income less than expenses	7 (4)	13 (12–28)	54 (40–74)	16 (10–25)	69 (67–75)^ab^	33 (31–35)^ab^
Income equal expenses	109 (62.3)	16 (4–41)	67 (12–84)	15 (7–35)	71 (30–75)^b^	32 (14–35)^b^
Income greater than expense	59 (33.7)	15 (5–36)	70 (30–84)	16 (7–35)	73 (47–75)^a^	34 (23–35)^a^
Test/*p* value	0.272/0.873^x^	3.382/0.184^x^	0.484/0.785^x^	7.375/**0.025**^x^	11.362/**0.003**^x^	
*Chronic disease*						
No	117 (66.9)	15 (4–41)	68 (12–84)	15 (7–32)	72 (30–75)	33 (14–35)
Yes	58 (33.1)	17 (4–36)	68 (21–84)	15 (7–35)	71 (54–75)	33 (23–35)
Test/*p* value	3794.5/0.202^y^	3489/0.761^y^	3397/0.990^y^	3317.5/0.809^y^	3665.5/0.382^y^	
*Continuous drug use during pregnancy*						
No	42 (24)	16 (5–41)	56.5 (33–84)	13,5 (7–30)	71,5 (30–75)	32,5 (14–35)
Yes	133 (76)	16 (4–36)	69 (12–84)	16 (7–35)	72 (47–75)	33 (23–35)
Test/*p* value		2737.5/0.846^y^	3356/**0.049**^y^	3171/0.186^y^	3035.5/0.393^y^	2829.5/0.897^y^
*Pregnancy type*						
Planned	122 (69.7)	16 (4–36)	69 (22–84)	15 (7–35)	72 (30–75)	33 (14–35)
Unplanned	53 (30.3)	15 (6–41)	61 (12–84)	16 (7–32)	73 (48–75)	33 (24–35)
Test/*p* value		2905/0.286^y^	2646/0.056^y^	3583/0.255^y^	3346.5/0.710^y^	3422.5/0.534^y^
*Pregnancy status*						
Primiparous	73 (41.7)	16 (4–35)	69 (12–84)	13 (7–35)	72 (47–75)	33 (18–35)
Multiparous	102 (58.3)	15.5 (4–41)	65.5 (21–84)	17 (7–35)	72 (30–75)	33 (14–35)
Test/*p* value		3789.5/0.840^y^	3317.5/0.219^y^	4440/**0.030**^**y**^	4238.5/0.116^y^	4223/0.126^y^

Statistically significant values (*p* < .05) are shown in bold

*FHAI* Fetal Health Anxiety Inventory, *MSPSS* Multidimensional Scale of Perceived Social Support

^x^Kruskall–Wallis H Test; ^y^Mann–Whitney U Test; ^a,b^There is no difference between groups with the same letter

The median scores of the scales used in this study were found to be 16 (range: 4–41) for the FHAI and 68 (range: 12–84) for the MSPSS. The median score for the anomie subdimension of the SWBS was found to be 15, for transcendence 72, and for harmony with nature 33. There was no statistically significant difference between the median FHAI scores and sociodemographic or obstetric variables (*p* > 0.05). However, the median MSPSS scores varied significantly by educational level (*p* = 0.035), with primary school graduates scoring a median of 60, high school graduates scoring 70, and university graduates scoring 71. Additionally, MSPSS scores differed significantly based on continuous medication use during pregnancy (*p* = 0.049). The median MSPSS score for those using medication was 69, while it was 56.5 for those not using medication (Table 1).

The anomie subdimension of the SWBS showed a significant difference regarding the pregnancy status (*p* = 0.030). Multiparous pregnant women had higher anomie scores compared to primiparous pregnant women. Moreover, the transcendence and harmony with Nature subdimensions of the SWBS exhibited significant differences by income level (*p* = 0.025 and *p* = 0.003, respectively). Pregnant women whose income exceeded their expenses had higher transcendence and harmony with nature scores when compared to those whose income was equal to their expenses (Table 1).

A positive and significant correlation was found between the anomie subdimension score and FHAI (*r* = 0.019, *p* < 0.001), as well as between the harmony with nature subdimension and the MSPSS (*r* = 0.223, *p* < 0.001) (Table 2).

**Table 2:** Descriptive statistics and correlation results of scale scores

	1	2	3	4	5	6	7	8
1. FHAI total score	1							
2. SWBS subdimension- Anomie	0.019**	1						
3. SWBS subdimension- Transcendence	− 0.014	− 0.131	1					
4. SWBS subdimension- Harmony with nature	− 0.027	− .163*	0.573**	1				
5. MSPSS total score	− 0.110	− 0.083	0.129	0.223**	1			
6.Family	− 0.046	− 0.106	0.077	0.126	0.515**	1		
7.Friends	− 0.126	− 0.089	0.135	0.281**	0.836**	0.389**	1	
8. Significant other	− 0.086	− 0.036	0.056	0.107	0.862**	0.346**	0.509**	1
*Descriptive statistics*								
Mean ± SD	16.5 ± 6.9	16.1 ± 6	69.9 ± 6.5	31.9 ± 3.3	63.6 ± 17	26 ± 3.8	19.2 ± 8	18.5 ± 9.2
Median (Minimum–Maximum)	16 (4–41)	15 (7–35)	72 (30–75)	33 (14–35)	68 (12–84)	28 (4–28)	21 (4–28)	22 (4–28)

*FHAI* Fetal Health Anxiety Inventory, *SWBS* Spiritual Well-Being Scale, *MSPSS* Multidimensional Scale of Perceived Social Support, *SD* Standard deviation

*< 0.05, **< 0.001

**Fig. 1:**
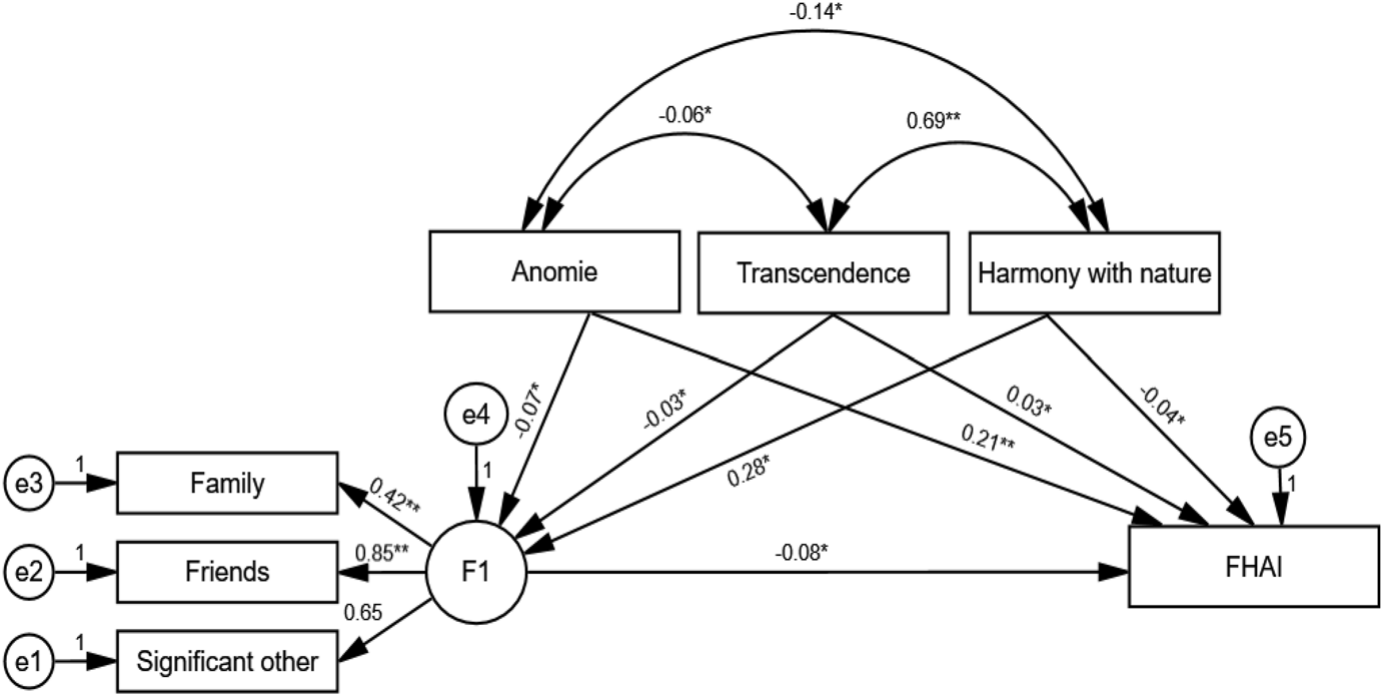
Path diagram. Standardized coefficient values are shown in the model. *Notes* * > 0.05, ** < 0.05; FHAI: Fetal Health Anxiety Inventory

To examine the relationships between fetal health anxiety, spiritual well-being, and perceived social support in high-risk pregnancies, structural equation modeling (SEM) was employed. The model fit indices were calculated as CMIN = 12.006, DF = 8, CMIN/DF = 1.501, RMSEA = 0.054, CFI = 0.981, and GFI = 0.980. Considering this model, an increase in the harmony with nature score resulted in a 0.282-unit increase in the MSPSS score (*β* = 0.282; *p* = 0.023), while an increase in the anomie subdimension score led to a 0.211-unit increase in the FHAI score (*β* = 0.211; *p* = 0.005) (Table 3) (Fig. 1).

**Table 3:** Path analysis results

			*β*^0^	*β*^1^	SE	Test value	*p*
*Structural Model*							
Family	<---	MSPSS	0.416	0.269	0.059	4.552	** < 0.001**
Friends	<---	MSPSS	0.849	1.145	0.237	4.825	** < 0.001**
Significant other	<---	MSPSS	0.648	1			
Anomie	<---	MSPSS	− 0.069	− 0.068	0.084	− 0.813	0.416
Transcendence	<---	MSPSS	− 0.032	− 0.030	0.105	− 0.282	0.778
Harmony with nature	<---	MSPSS	0.282	0.513	0.226	2.277	**0.023**
FHAI	<---	MSPSS	− 0.082	− 0.094	0.102	− 0.925	0.355
Anomie	<---	FHAI	0.211	0.241	0.085	2.828	**0.005**
Transcendence	<---	FHAI	0.034	0.036	0.107	0.331	0.741
Harmony with nature	<---	FHAI	− 0.036	− 0.076	0.221	− 0.343	0.732

Statistically significant values (*p* < .05) are shown in bold

*β*^*0*^ Standardized beta coefficient, *β*^*1*^ Unstandardized beta coefficient, *SE* Standard error, *FHAI* Fetal Health Anxiety Inventory, *MSPSS* Multidimensional Scale of Perceived Social Support

## Discussion

This study examined the relationships between fetal health anxiety, spiritual well-being, and perceived social support among women with high-risk pregnancies. Fetal health anxiety is one of the most common concerns; expectant mothers have regarding the health of their fetus (Reiser & Wright, 2019). The total score for fetal health anxiety was determined to be 16 in the sample of the present study. Previous studies reported lower levels of fetal health anxiety (İbici Akca et al., 2024; Reiser & Wright, 2019; Sabancı Baransel & Barut, 2024). This discrepancy may be because the study group consisted of women diagnosed with high-risk pregnancies.

In this study, the perceived social support score among participants was reported to be 68, which was found to be higher than the scores reported by Kaydırak et al. (2024) in their study on pregnant women diagnosed with fetal anomalies. During hospitalization, increased attention and support from family and friends may have strengthened the physical and emotional support mechanisms, thereby enhancing the perception of social support among these women. Sociodemographic analyses indicated that pregnant women with higher levels of education and those who were on continuous medication had higher perceived social support levels. The finding that women with higher education levels reported a higher level of perceived social support is consistent with the study carried out by Baharvand et al. (2022). Educated women may effectively utilize social support networks and exhibit greater confidence in seeking support due to better access to information and advanced communication skills. Moreover, women who used medication continuously during pregnancy were determined to have a higher level of perceived social support. Continuous medication use often necessitates heightened attention and care for health management, which could lead to greater social support from the individual’s surroundings. The increased need for care and health-related concerns may motivate these women to actively seek and receive support from others.

Spiritual well-being provides significant support in coping with the challenges and stress caused by illnesses, positively influencing individuals’ health (Akbarzadeh & Ahmadinezhad, 2019). Among the pregnant women who participated in this study, the anomie subdimension of the SWBS was found to differ based on pregnancy status. It was determined that multiparous pregnant women had higher levels of Anomie. Anomie refers to situations in which the social order is disrupted, causing individuals to become detached from social norms and values, leading to existential void and loss of direction (Ryff, 2021). Hospitalization, particularly for multiparous pregnant women, may contribute to role conflicts due to concerns related to health problems, separation from their social environment, and the inability to provide sufficient support to their other children. This situation may make it more difficult for pregnant women to adhere to social norms and roles, leading to heightened feelings of alienation. Harmony with nature is an integral component of spiritual well-being, encompassing an individual’s awareness of and adaptation to their physical environment. Transcendence, on the other hand, refers to belief in a powerful and eternal entity and the recognition of one’s own vulnerability and dependence on that entity (Ekşi & Kardaş, 2017; Fisher, 2003). Among the pregnant women in the present study, the transcendence and harmony with nature subdimensions of the SWBS were found to differ in terms of income level. Pregnant women whose income exceeded their expenses exhibited higher levels of transcendence and harmony with nature in comparison with those whose income is equal to their expenses. This result suggests that economic stability may enhance spiritual experiences and an individual’s relationship with their environment. A favorable economic situation is thought to contribute to reduced financial concerns regarding the baby, the timely fulfillment of needs, inner peace, and an increased capacity to adapt to one’s surroundings.

In this study, a negative relationship was found between spiritual well-being and fetal health anxiety among pregnant women. This result suggests that spiritual well-being could be an effective factor in reducing pregnant women’s concerns about fetal health. Nourimand et al. (2020) indicated in their study that pregnant women with higher levels of spiritual well-being exhibit better mental health and experience fewer negative emotions. Similarly, the study carried out by Oktafia et al. (2021) revealed a negative relationship between the level of spiritual well-being and anxiety in pregnant women. Studies examining the relationship between spirituality and health may sometimes yield tautological (self-referential) findings (Koenig & Carey, 2024, 2025). In this context, the positive association between the anomie subdimension of the SWBS and spiritual well-being suggests that this relationship may be associated with psychological constructs. Therefore, when assessing the anxiety-reducing effect of spiritual well-being, its interaction with psychological structures should be considered more carefully.

Unlike received social support, perceived social support refers to the support individuals believe is available to them (Feng et al., 2024). As determined in this study, the perceived social support score also increases together with an increase in level of harmony with nature. This study revealed a positive relationship between perceived social support and spiritual well-being. Similarly, previous studies reported a positive association between the level of perceived social support and spiritual well-being among pregnant women (Akbarzadeh & Ahmadinezhad, 2019; Niaghiha et al., 2019). Spiritual well-being helps individuals build strong relationships with themselves, God, and their environment (Tirgari et al., 2022). Individuals who experience a higher level of harmony with nature may develop a deeper sense of being part of the creation within their environment, thereby strengthening their perception of social support. This factor could be particularly significant in the recovery process of vulnerable groups, such as hospitalized women with high-risk pregnancies. Therefore, the integration not only of medical interventions but also of social and spiritual support into the treatment processes of pregnant women is crucial.

## Limitations

This study has several limitations. First, as the research was conducted in a single hospital, the data obtained may only represent the sample group and cannot be generalized. Second, the exclusive use of self-report scales completed solely by participants is thought to have increased method variance. The positive relationship between the Anomie subscale of the SWBS and the FHAI may be linked to underlying psychological structures. Therefore, the analyses were conducted based on the subscales of the measurement tools, and the results should be interpreted carefully.

## Conclusion

It was determined in this study that hospitalized women diagnosed with high-risk pregnancies exhibited low levels of fetal health anxiety, and high levels of perceived social support. While harmony with nature was found to improve perceived social support, anomie was associated with increased fetal health anxiety. Furthermore, perceived social support levels were found to vary based on sociodemographic variables such as education level and continuous medication use. The anomie subscale of the SWBS showed significant differences based on pregnancy status, while the transcendence and harmony with nature subscales varied by income level. These results emphasize that healthcare professionals caring for women with high-risk pregnancies should not only focus not only on medical interventions but also on providing spiritual support and strengthening social support networks. The integration of a holistic approach into clinical practice could be a significant step toward improving both the physical and psychological health of pregnant women.

## Data Availability

The datasets of the current study are available from the corresponding author on reasonable request.

## References

[CR1] Akbari, V., Rahmatinejad, P., Shater, M. M., Vahedian, M., & Khalajinia, Z. (2020). Investigation of the relationship of perceived social support and spiritual well-being with postpartum depression. *Journal of Education and Health Promotion,**9*, 174. 10.4103/jehp.jehp_56_20 32953903 10.4103/jehp.jehp_56_20 PMC7482628

[CR2] Akbarzadeh, M., & Ahmadinezhad, F. (2019). Investigating the relationship of spiritual wellbeing with perceived stress and perceived social support among women with preeclampsia. *Health, Spirituality and Medical Ethics,**6*, 2–9. 10.29252/jhsme.6.4.2

[CR3] Baharvand, P., Anbari, K., & Hamidi, H. (2022). Perceived social support in pregnant women with gestational diabetes attending hospitals in western Iran compared to healthy controls and its relationship with perceived anxiety. *Journal of Diabetes & Metabolic Disorders,**21*(2), 1549–1555. 10.1007/s40200-022-01100-5 36404846 10.1007/s40200-022-01100-5 PMC9672204

[CR4] Baltacı, N., & Vatansever, S. (2024). Investigation of distress and tolerance to distress in risky pregnant women. *Journal of General Health Sciences,**6*(2), 318–330.

[CR5] Bedaso, A., Adams, J., Peng, W., & Sibbritt, D. (2021). The relationship between social support and mental health problems during pregnancy: a systematic review and meta-analysis. *Reproductive Health,**18*(1), 162. 10.1186/s12978-021-01209-5 34321040 10.1186/s12978-021-01209-5 PMC8320195

[CR6] Bilgiç, G., & Çıtak Bilgin, N. (2021). Relationship between fear of childbirth and psychological and spiritual well-being in pregnant women. *Journal of Religion Health,**60*(1), 295–310. 10.1007/s10943-020-01087-4 32949330 10.1007/s10943-020-01087-4

[CR7] Chehrazi, M., Faramarzi, M., Abdollahi, S., Esfandiari, M., & Shafie Rizi, S. (2021). Health promotion behaviours of pregnant women and spiritual well-being: Mediatory role of pregnancy stress, anxiety and coping ways. *Nursing Open,**8*(6), 3558–3565. 10.1002/nop2.905 33938639 10.1002/nop2.905 PMC8510764

[CR8] Eker, D., & Arkar, H. (1995). Çok boyutlu algılanan sosyal destek ölçeğinin faktör yapısı, geçerlik ve güvenirliği. *Türk Psikoloji Dergisi,**10*(34), 45–55.

[CR9] Ekşi, H., & Kardaş, S. (2017). Spiritual well-being: Scale development and validation. *Spiritual Psychology and Counseling,**2*(1), 73–88. 10.12738/spc.2017.1.0022

[CR10] Feng, Y., Liu, X., Zhang, S., Lin, T., Guo, X., & Chen, J. (2024). Relationship among post-traumatic growth, spiritual well-being, and perceived social support in Chinese women with gynecological cancer. *Scientific Reports,**14*(4847), 1–9. 10.1038/s41598-024-55605-5 38418533 10.1038/s41598-024-55605-5 PMC10902294

[CR11] Gökbulut, N., Bal, Z., & Ucar, T. (2024). Fetal health anxiety: A validity and reliability study of the Turkish version of the Fetal health anxiety inventory. *Bezmialem Science,**12*, 107–118. 10.14235/bas.galenos.2023.28863

[CR12] Gomez, R., & Fisher, J. W. (2003). Domains of spiritual well-being and development and validation of the Spiritual Well-Being Questionnaire. *Personality and Individual Differences,**35*(8), 1975–1991.

[CR13] Gürbüz, S. (2019). *Yapısal eşitlik modellemesinin kuramsal temelleri. In AMOS ile yapısal eşitlik modellemesi*. Seçkin Yayıncılık.

[CR14] Hosaini, S., Yazdkhasti, M., Moafi Ghafari, F., Mohamadi, F., Kamran Rad, S. H. R., & Mahmoodi, Z. (2023). The relationships of spiritual health, pregnancy worries and stress and perceived social support with childbirth fear and experience: A path analysis. *PLoS ONE,**18*(12), e0294910. 10.1371/journal.pone.0294910 38060610 10.1371/journal.pone.0294910 PMC10703247

[CR15] İbici Akca, E., Gökbulut, N., & Cengizhan, S. O. (2024). The effects of MBSR programme on prenatal comfort and fetal health anxiety in pregnant women. *Journal of Reproductive and Infant Psychology,**42*(3), 449–463. 10.1080/02646838.2023.2227219 37342975 10.1080/02646838.2023.2227219

[CR16] Isaacs, N. Z., & Andipatin, M. G. (2020). A systematic review regarding women’s emotional and psychological experiences of high-risk pregnancies. *BMC Psychology,**8*(45), 1–11. 10.1186/s40359-020-00410-8 32362285 10.1186/s40359-020-00410-8 PMC7197168

[CR17] Karakoç, H., Özkan, H., Kanbur, A., & Aksoy, A. N. (2023). The relationship between distress and prenatal attachment during pregnancy. *Journal of General Health Sciences,**5*(2), 201–207.

[CR18] Kardaş, S. (2019). Erratum: Correcting the name of the spiritual well-being scale as the three-factor spiritual well-being scale. *Spiritual Psychology and Counseling,**4*(1), 85–85. 10.12738/spc.2019.4.1.0068

[CR19] Kaydırak, M. M., Balkan, E., Bacak, N., & Kızoglu, F. (2024). Perceived social support and depression, anxiety and stress in pregnant women diagnosed with foetal anomaly. *Journal of Advanced Nursing*. 10.1111/jan.16587 39494755 10.1111/jan.16587 PMC12271646

[CR20] Koenig, H. G., & Carey, L. B. (2024). Religion, spirituality and health research: Warning of contaminated scales. *Journal of Religion and Health,**63*(5), 3729–3743. 10.1007/s10943-024-02112-6 39196443 10.1007/s10943-024-02112-6

[CR21] Koenig, H. G., & Carey, L. B. (2025). Approaches for analyzing the relationship between spirituality and health using measures contaminated with indicators of mental and social health. *Journal of Religion and Health*. 10.1007/s10943-025-02249- 39808227 10.1007/s10943-025-02249-y

[CR22] Niaghiha, M., Baglooei, M. M., Mafi, M., & Taherpour, M. (2019). Spiritual well-being and life satisfaction in pregnant women: the mediating role of social support. *Asian Journal of Social Health and Behavior,**2*(3), 83–88.

[CR23] Nourimand, F., Forouhari, S., Jahromi, B. N., Asadi, N., Abbasi, Z., Beygi, Z., Karimi, M., & Sarbakhsh, F. (2020). Studying the correlation between spiritual well-being and religious attitude with mental health and quality of life in pregnant women. *Pakistan Journal of Medical and Health Sciences,**14*, 681–684.

[CR24] Oktafia, R., Indriastuti, N. A., & Kusuma, A. N. (2021). Association between spiritual well-being and anxiety among high-risk pregnant women. *Bali Medical Journal,**10*(3), 1375–1378. 10.15562/bmj.v10i3.3055

[CR25] Ozbay, F., Johnson, D. C., Dimoulas, E., Morgan, C. A., Charney, D., & Southwick, S. (2007). Social support and resilience to stress: From neurobiology to clinical practice. *Psychiatry (Edgmont),**4*(5), 35–40. 20806028 PMC2921311

[CR26] Palma, E., Armijo, I., Cifuentes, J., Ambiado, S., Rochet, P., Díaz, B., Gutierrez, J., & Mena, C. (2021). Hospitalisation in high-risk pregnancy patients: Is prenatal attachment affected? *Journal of Reproductive and Infant Psychology,**39*(1), 30–42. 10.1080/02646838.2020.1740661 32223427 10.1080/02646838.2020.1740661

[CR27] Reiser, S. J. (2019). *Examining health anxiety and anxiety about fetal health during pregnancy*. The University of Regina.

[CR28] Reiser, S. J., & Wright, K. D. (2019). Fetal health anxiety: Development and psychometric properties of the fetal health anxiety inventory. *Journal of Psychosomatic Obstetrics & Gynecology,**40*(4), 264–273. 10.1080/0167482x.2018.1490722 30089227 10.1080/0167482X.2018.1490722

[CR29] Ryff, C. D. (2021). Spirituality and well-being: Theory, science, and the nature connection. *Religions (Basel),**12*(11), 1–19. 10.3390/rel12110914 10.3390/rel12110914 PMC8651234 34881052

[CR30] Sabancı Baransel, E., & Barut, S. (2024). Effect of diaphragmatic breathing exercises on fetal health anxiety and coping with prenatal stress. *Journal of Exercise Therapy & Rehabilitation,**11*(2), 81–89. 10.15437/jetr.1383622

[CR31] Sarmasti, N., Ayoubi, S. H., Mahmoudi, G., & Heydarpour, S. (2019). Comparing perceived social support and perceived stress in healthy pregnant women and pregnant women with preeclampsia. *Ethiopian Journal of Health Sciences,**29*(3), 369–376. 10.4314/ejhs.v29i3.9 31447505 10.4314/ejhs.v29i3.9 PMC6689728

[CR32] Tirgari, B., Khaksari, M., Soltani, Z., Mirzaee, M., Saberi, S., & Bashiri, H. (2022). Spiritual well-being in patients with chronic diseases: A systematic review and meta-analysis. *Journal of Religion and Health,**61*(5), 3969–3987. 10.1007/s10943-022-01595-5 35794504 10.1007/s10943-022-01595-5

[CR33] Zimet, G. D., Dahlem, N. W., Zimet, S. G., & Farley, G. K. (1988). The multidimensional scale of perceived social support. *Journal of Personality Assessment,**52*(1), 30–41. 10.1207/s15327752jpa5201_2 10.1080/00223891.1990.9674095 2280326

